# Mothers’ Knowledge of and Practices Toward Oral Hygiene of Children Aged 5-9 Years in Bangladesh: Cross-Sectional Study

**DOI:** 10.2196/59379

**Published:** 2025-02-03

**Authors:** Tahazid Tamannur, Sadhan Kumar Das, Arifatun Nesa, Foijun Nahar, Nadia Nowshin, Tasnim Haque Binty, Shafiul Azam Shakil, Shuvojit Kumar Kundu, Md Abu Bakkar Siddik, Shafkat Mahmud Rafsun, Umme Habiba, Zaki Farhana, Hafiza Sultana, Anton Abdulbasah Kamil, Mohammad Meshbahur Rahman

**Affiliations:** 1 Department of Health Education, National Institute of Preventive and Social Medicine, Dhaka, Bangladesh; 2 Department of Public Health and Hospital Administration, National Institute of Preventive and Social Medicine, Mohakhali, Dhaka, Bangladesh; 3 Directorate General of Health Services, Ministry of Health & Family Welfare, Government of the People's Republic of Bangladesh, Dhaka, Bangladesh; 4 School of the Environment, Nanjing University, Nanjing, China; 5 Dental Speciality Center, Dhaka, Bangladesh; 6 BRAC James P Grant School of Public Health, BRAC University, Dhaka, Bangladesh; 7 Credit Information Bureau, Bangladesh Bank, Dhaka, Bangladesh; 8 Department of Business Administration, Istanbul Gelisim University, Istanbul, Turkey; 9 Department of Biostatistics, National Institute of Preventive and Social Medicine, Dhaka, Bangladesh

**Keywords:** mothers’ knowledge and practices, oral hygiene, child oral health, Bangladesh

## Abstract

**Background:**

Healthy oral hygiene is crucial for overall health and well-being. Parents’ dental care knowledge and practices affect their children’s oral health.

**Objective:**

This study examined mothers’ knowledge and practices regarding their children’s oral hygiene through a cross-sectional survey.

**Methods:**

This cross-sectional survey was conducted from January 1 to December 31, 2022, in Dhaka, Bangladesh. Mothers’ knowledge and practices regarding their children’s oral hygiene were assessed through a semistructured questionnaire. Statistical analyses, including the *χ*^2^ test and Pearson correlation test, were performed. The Mann-Whitney *U* and Kruskal-Wallis 1-way ANOVA tests were also used to show the average variations in knowledge and practices among different sociodemographic groups.

**Results:**

Of 400 participants, the mean age of mothers was 30.94 (SD 5.15) years, and 388 (97%) were of the Muslim faith, 347 (86.8%) were housewives, and 272 (68%) came from nuclear families. A total of 165 (41.3%) participants showed good knowledge of their children’s oral hygiene, followed by 86 (21.5%) showing moderately average knowledge, 75 (18.8%) showing average knowledge, and 74 (18.5%) showing poor knowledge. A total of 182 (45.5%) mothers had children with good oral hygiene practices, followed by mothers with children who had average (n=78, 19.5%), moderately average (n=75, 18.8%), and poor (n=65, 16.3%) oral hygiene practices. The mother’s knowledge level was significantly associated with age (*P*=.01), education (*P*<.001), family size (*P*=.03), and monthly income (*P*<.001). On the other hand, educational status (*P*=.002) and income (*P*=.04) were significantly associated with the mother’s practices regarding their children’s oral hygiene. Nonparametric analysis revealed that mothers who were older (mean knowledge score: 12.13, 95% CI 10.73-13.54 vs 11.21, 95% CI 10.85-11.58; *P*=.01), with a bachelor’s degree or higher (mean knowledge score: 12.93, 95% CI 12.55‐13.31 vs 9.66, 95% CI 8.95‐10.37; *P*<.001), who were working mothers (mean knowledge score: 12.30, 95% CI 11.72‐12.89 vs 11.45, 95% CI 11.17‐11.73; *P*=.03), and who had a higher family income (mean knowledge score: 12.49, 95% CI 12.0‐12.98 vs 10.92, 95% CI 10.48‐11.36; *P*<.001) demonstrated significantly higher levels of oral health knowledge. Conversely, good oral hygiene practices were significantly associated with higher maternal education (mean practice score: 6.88, 95% CI 6.54‐7.22 vs 6.01, 95% CI 5.63‐6.40; *P*<.001) and family income (mean practice score: 6.77, 95% CI 6.40‐7.14 vs 5.96, 95% CI 5.68‐6.24; *P*=.002). The mother’s knowledge was also significantly and positively correlated (Pearson correlation coefficient *r*=0.301; *P*<.001) with their children’s oral hygiene practices, shown by both the Pearson chi-square (*χ*^2^=25.2; *P*<.001) test and correlation coefficient.

**Conclusions:**

The mothers’ knowledge and their children’s oral hygiene practices were inadequate. The mother’s age, education level, family size, and monthly income significantly influenced their knowledge level. Children’s oral hygiene habits were significantly associated with family income and the mother’s educational status. This underscores the need for educational programs, accessible dental care services, oral health education in the curriculum, media and technology involvement in oral health educational campaigns, and proper research and monitoring.

## Introduction

According to the World Health Organization, dental caries, periodontal disease, tooth loss, mouth cancer, oro-dental trauma, noma, and congenital defects including cleft lip and palate are classified as oral diseases. Oral health issues are prevalent in low-income nations owing to poor socio–educational-economic circumstances [1]. In terms of general health and well-being, there is a significant connection between oral health and overall health [2,3]. It impacts individuals’ capacity to do tasks, communicate, and engage in social interactions. Thus, it has an impact on both the physical and psychological aspects of an individual [4]. Most common oral health problems and conditions can be readily avoided by establishing suitable oral hygiene routines, such as twice daily brushing with the best toothbrush, using fluoride-containing toothpaste, and using the proper brushing technique [5]. Other preventive measures include eating a balanced diet low in free sugar, going to the dentist regularly for exams, and receiving treatment for illnesses when they are still in the early stages [6]. It can be minimized by practicing good oral hygiene habits, such as brushing and flossing teeth and visiting the dentist frequently [7].

Worldwide, over 2 billion individuals have dental caries in their permanent teeth, while 514 million children have dental caries in their primary teeth [8]. Early childhood caries (ECC) in children have been linked mostly to poor dental hygiene. Infants and toddlers with significant plaque accumulation were more likely to experience severe ECC and caries from birth to toddlerhood [9]. ECC has several causes, including excessive sugar intake, poor dental hygiene, inadequate fluoride exposure, and enamel abnormalities [10]. So, the development of caries and the acquisition of infection are substantially influenced by diet and feeding habits.

The children in Bangladesh have various infections and disorders [11-13]. Poor oral health is another prevalent health problem among them, which is still neglected [13]. As parents are the major caregivers, their involvement is crucial in the maintenance and development of excellent oral health in children, such as teaching healthy eating and drinking habits [14]. In addition, several factors impact the dental health of children, including the mother’s level of education, the mother’s work situation, and her understanding of oral hygiene [15]. The adoption of good oral health practices in children is influenced by the parents’, and particularly the mother’s, oral health knowledge, attitudes, and awareness [16]. An Indian study found that the oral hygiene quality of children aged 12 years was shown to be significantly influenced by their mother’s oral hygiene knowledge [17]. Children with high rates of dental caries and low rates of fillings were found to have parents with inadequate oral health literacy, according to another study [18]. As a result, it is essential for parents, and particularly mothers, to have awareness about oral health. Scholars argued that a mother’s knowledge about oral health and the consequence of adequate dental hygiene has a beneficial impact on their children’s dental well-being and adherence to dental care practices [19,20].

Research on dental caries awareness among parents in Pakistan has found low levels of knowledge about oral hygiene standards [21]. A study conducted in India on the oral health status of children aged 3‐6 years and their mother’s oral health–related knowledge, attitude, and practices found most mothers had a medium level of knowledge, an average level of attitude, and a high level of practices regarding oral health [22]. Another study in Malaysia on parental knowledge and practices in preschool children’s oral health found that the majority had good knowledge [23]. Numerous studies have been conducted globally regarding parents’ or mothers’ oral hygiene knowledge and practices, but these have been insufficient, particularly among mothers in Bangladesh. There is a lack of research investigating the extent to which mothers are aware of and follow oral hygiene practices. Hence, this study aimed to assess mothers’ level of oral hygiene knowledge and practices regarding their 5- to 9-year-old children.

## Methods

This study followed the STROBE (Strengthening the Reporting of Observational Studies in Epidemiology) guidelines to prepare the manuscript, and the STROBE checklist is provided in Multimedia Appendix 1.

### Ethical Considerations

Permission to conduct this study was given by the institutional review board of the National Institute of Preventive and Social Medicine (NIPSOM), Bangladesh (Ref NIPSOM/IRB/2017/09). The Shaheed Suhrawardy Medical College Hospital and Dhaka Dental College Hospital provided the necessary documentation. Both written and verbal consent were taken before initiating the interview. Participants received an overview of the study’s goals, and those who consented were eventually included. No compensation was given to the participants, and data anonymity was strictly maintained.

### Study Setting and Participants

This cross-sectional study was conducted from January 1 to December 31, 2022, in two tertiary-level hospitals in Dhaka South named Shaheed Suhrawardy Medical College Hospital and Dhaka Dental College Hospital in Dhaka City. Mothers of children aged 5-9 years who visited these tertiary hospitals were interviewed through a semistructured questionnaire.

### Study Pretesting

To observe the overall scenario including questionnaire information, possible sampling techniques, and approximate nonresponse rate in the study, we first performed a pretest of the study. The pretesting was conducted among 50 mothers of children aged 5‐9 years in the Sapporo Dental College & Hospital located at Dhaka North.

### Sampling Technique and Sample Size

A convenience sampling technique was followed for this study. During the literature search, no study was found that assessed the knowledge and practices toward children’s oral hygiene among Bangladeshi mothers. However, a study was found from India with a similar sociodemography. Mohandass et al [20] showed that the prevalence of adequate knowledge and practices were 58% and 57%, respectively. The sample size for this study was calculated using the below equation.


(1) $$n=\frac{z^{2}pq}{d^{2}}$$

The sample size when *P*=.58 for the mother’s knowledge was:


$$n=\frac{1.96^{2}\times 0.58\times \left(1-0.58\right)}{0.05^{2}}=375$$

Similarly, the sample size when *P*=.57 for the mother’s practice level was:


$$n=\frac{1.96^{2}\times 0.57\times \left(1-0.57\right)}{0.05^{2}}=377$$

Therefore, we initially chose a maximum of 377 as the required sample size. Considering a maximum 5% nonresponse rate (based on pretesting), we rounded up this figure and selected 400 as an approximate sample size for the study.

### Selection Criteria

The inclusion criteria for this study were mothers of Bangladeshi nationality who were living in Dhaka for at least 1 year, mothers of children aged 5‐9 years, and mothers who provided consent and agreed to participate in the study. The exclusion criteria for the study were mothers who were not Bangladeshi but currently living in Dhaka, mothers of children older than 10 years or younger than 5 years, and mothers younger than 21 years or older than 48 years.

### Sociodemographic Variables

Respondents’ sociodemographic variables such as age (21-48 years), religion (Muslim, non-Muslim), educational status (up to primary, secondary, higher secondary, and bachelor’s degree or higher), occupational status (housewife, working), family type (nuclear, joint), family size (<5 persons, ≥5 persons), and monthly family income (≤20,000 BDT, 20,001‐40,000 BDT, ≥40,001 BDT; a currency exchange rate of 101.85 BDT=US $1 was used) were the independent variables in this study.

### Measurement of Knowledge and Practice

The study used 15 variables to assess mothers’ knowledge and 13 to assess their children’s practices related to oral hygiene (MultimediaAppendices 2  3). Both knowledge and practice questions were adopted from the existing literature and revised according to our selection criteria. The summation scoring technique was used in computation, and the descriptive statistics, including percentiles, were observed. The range for the knowledge and practice scores were 1-15 and 1-13, respectively. According to the percentile approach, knowledge was classified into four levels: poor (<25% percentile cut point: ≤9.999), moderately average (25%‐49% percentile cut point: 10.0‐11.99), average (50%‐74% percentile cut point: 12.0‐12.99), and good knowledge (≥75% percentile cut point: ≥13.0) [24]. Practices were also classified into four levels: poor (<25% percentile cut point:≤4.99), moderately average (25%‐49% percentile cut point: 5.0‐5.999), average (50%‐74% percentile cut point: 6.0‐6.99), and good practices (≥75% percentile cut point: ≥7.0). For all cases, the cut points were statistically evident [25,26].

### Data Quality Control

To ensure the reliability and validity of the study findings, we observed the reliability analysis for both knowledge and practice variables, yielding a Cronbach α; the reliability coefficient values for the variables related to knowledge and practice were found to be 0.78 and 0.81, indicating acceptable internal consistency.

### Statistical Analysis

Descriptive statistics were performed to present participants’ sociodemographic characteristics and mean knowledge and practice scores. The Pearson *χ*^2^ test and Pearson correlation coefficient were used as a bivariate analysis. Since both knowledge and practice scores did not follow normality, we performed the Mann-Whitney *U* test and Kruskal-Wallis 1-way ANOVA test to show the mean knowledge and practice score variations between two (eg, housewife vs working mother) and more than two groups (eg, different age groups), respectively. Necessary assumptions were checked before performing the statistical analysis. All the data management and statistical analyses were carried out through SPSS Statistics 27.0 (IBM Corp). The *P* value was observed for all the cases at a 5% level, and 95% was considered as the CI [27-29].

## Results

### Sociodemographic Characteristics of the Respondents

The majority of the respondents (n=209, 52.3%) were within the 21‐30 years age group, followed by 44% (n=176) in the 31‐40 years age group. Most (n=57, 39.3%) respondents had a secondary level of education. Most were Muslims (n=388, 97%) and housewives (n=347, 86.8%). Many of the respondents (n=157, 39.3%) had a monthly family income of 20,001‐40,000 BDT (US $206.19-$392.73) per month. About 13.3% (n=53) of mothers were working (Table 1).

**Table 1.** Distribution of sociodemographic characteristics of the respondents (N=400).

Characteristics	Respondents, n (%)
Age group (years)	
21‐30	209 (52.3)
31‐40	176 (44.0)
41‐48	15 (3.8)
Religion	
Muslim	388 (97.0)
Non-Muslim	12 (3.0)
Educational status	
Up to primary	76 (19.0)
Secondary	157 (39.3)
Higher secondary	68 (17.0)
Bachelor’s degree or higher	99 (24.8)
Occupation	
Housewife	347 (86.8)
Working	53 (13.3)
Family type	
Nuclear	272 (68.0)
Joint	128 (32.0)
Number of family members	
<5 persons	193 (48.3)
≥5 persons	207 (51.8)
Monthly family income (BDT)^a^	
≤20,000	143 (35.8)
20,001‐40,000	157 (39.3)
≥40,001	100 (25.0)

a A currency exchange rate of 101.85 BDT=US $1 was used.

### Knowledge Among Mothers Regarding Their Children’s Oral Hygiene

Multimedia Appendix 4 shows the mothers’ knowledge scores regarding their children’s oral hygiene. Among the 400 mothers, more than 90% (n=360) knew the importance of brushing teeth, while 82.3% (n=329) and 80.8% (n=323) knew the recommended frequency and appropriate time for brushing teeth, respectively. Surprisingly, only 29.5% (n=118) and 38.5% (n=154) knew the duration for brushing teeth and that fluoride protects against caries, respectively. However, most of the respondents knew about the “importance of cleaning tongue” (n=365, 91.3%), “gingival disease common cause of gum bleeding” (n=286, 71.5%), “brushing and flossing protect against bleeding gum” (n=243, 60.8%), “yellow coating plaque” (n=362, 90.5%), “sugary item cause caries” (n=387, 96.8%), “soft drinks cause caries” (n=295, 73.8%), and “regular brushing protects against caries” (n=380, 95%).

### Mothers’ Practices Regarding Their Children’s Oral Hygiene

Multimedia Appendix 5 shows the individual distribution of mothers’ practices regarding their children’s oral hygiene. Most (n=381, 95.3%) of the mothers reported that their child brushed their teeth regularly, 99% (n=396) of children used a toothbrush, 62% (n=248) changed their toothbrush every 3‐4 months or if the bristles were frayed, 97.8% (n=391) used their toothpaste, and 77.8% (n=311) rinsed their mouth after eating. Surprisingly, 44.3% (n=177) of children brushed their teeth twice daily, 42% (n=168) cleaned their tongues, and 2.8% (n=11) used floss. Only 12.5% (n=50) were given sugary items with meals, and 0.3% (n=1) were taken to dentists every 6 months.

### Overall Knowledge and Practice Levels of the Respondents

**Figure 1.**
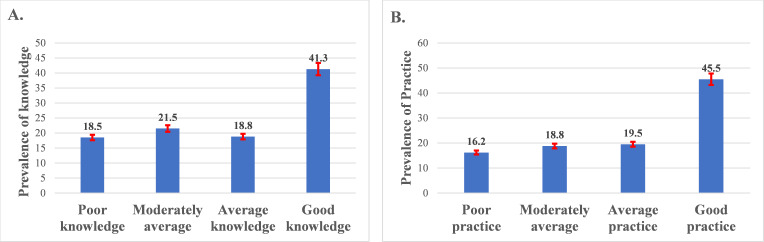
(A) Distribution of the overall knowledge of mothers. (B) Distribution of the overall practices of mothers.

Figure 1 depicts the level of knowledge and practices of mothers regarding their children’s oral hygiene and the association with the mother’s educational status. Only 41.3% (n=165) had good knowledge, while 18.5% (n=74) had poor knowledge (Figure 1A). Similarly, only 45.5% (n=182) of the mothers showed good practices, while 16.2% (n=65) showed poor practice levels (Figure 1B).

### Sociodemographic Variations in the Mother’s Knowledge Level Regarding Their Children’s Oral Hygiene

A total of 66.7% (10/15) of mothers aged 41-48 years had good knowledge regarding their children’s oral hygiene. The Pearson *χ*^2^ association test revealed that mothers’ knowledge levels were significantly associated with age (*P*=.01), education (*P*<.001), family size (*P*=.03), and monthly income (*P*<.001; Table 2).

**Table 2.** Association of mothers’ knowledge with sociodemographic characteristics.

Characteristics	Poor knowledge, n (%)	Moderately average, n (%)	Average knowledge, n (%)	Good knowledge, n (%)	*P* value^a^
Age group (years)					.01
21‐30 (n=209)	43 (20.6)	55 (26.3)	39 (18.7)	72 (34.4)	
31‐40 (n=176)	28 (15.9)	29 (16.5)	36 (20.5)	83 (47.2)	
41‐48 (n=15)	3 (20.0)	2 (13.3)	0 (0.0)	10 (66.7)	
Religion					.22
Muslim (n=388)	72 (18.6)	86 (22.2)	72 (18.6)	158 (40.7)	
Non-Muslim (n=12)	2 (16.7)	0 (0.0)	3 (25.0)	7 (58.3)	
Educational status					<.001
Up to primary (n=76)	32 (42.1)	22 (28.9)	8 (10.5)	14 (18.4)	
Secondary (n=157)	29 (18.5)	46 (29.3)	33 (21.0)	49 (31.2)	
Higher secondary (n=68)	8 (11.8)	8 (11.8)	16 (23.5)	36 (52.9)	
Bachelor’s degree or higher (n=99)	5 (5.1)	10 (10.1)	18 (18.2)	66 (66.7)	
Occupation					.10
Housewife (n=347)	68 (19.6)	77 (22.2)	67 (19.3)	135 (38.9)	
Working (n=53)	6 (11.3)	9 (17.0)	8 (15.1)	30 (56.6)	
Family type					.06
Nuclear (n=272)	46 (16.9)	52 (19.1)	59 (21.7)	115 (42.3)	
Joint (n=128)	28 (21.9)	34 (26.6)	16 (12.5)	50 (39.1)	
Number of family members					.03
<5 persons (n=193)	27 (14.0)	36 (18.7)	39 (20.2)	91 (47.2)	
≥5 persons (n=207)	47 (22.7)	50 (24.2)	36 (17.4)	74 (35.7)	
Monthly family income (BDT)^b^					<.001
≤20,000 (n=143)	38 (26.6)	37 (25.9)	25 (17.5)	43 (30.1)	
20,001‐40,000 (n=157)	26 (16.6)	33 (21.0)	37 (23.6)	61 (38.9)	
≥41,001 (n=100)	10 (10.0)	16 (16.0)	13 (13.0)	61 (61.0)	

a *χ*2/Fisher exact test.

b A currency exchange rate of 101.85 BDT=US $1 was used.

### Sociodemographic Variation of the Mother’s Practice Level Regarding Their Children’s Oral Hygiene

Table 3 represents the association between mothers’ sociodemographic characteristics and their practices regarding their children’s oral hygiene. The analysis found that more than half (n=8, 53.3%) of older-aged mothers had good practices, and 66.7% (n=60) of mothers with a bachelor’s degree or higher showed good practices regarding their children’s oral hygiene. The educational status (*P*=.002) and income (*P*=.04) were significantly associated with the mothers’ practices regarding their children’s oral hygiene (Table 3).

**Table 3.** Association between sociodemographic characteristics and practice level regarding their children’s oral hygiene.

Characteristics	Poor practice, n (%)	Moderately average, n (%)	Average practice, n (%)	Good practice, n (%)	*P* value^a^
Age group (years)					.34
21‐30 (n=209)	34 (16.3)	44 (21.1)	46 (22.0)	85 (40.7)	
31‐40 (n=176)	30 (17.0)	27 (15.3)	30 (17.0)	89 (50.6)	
41‐48 (n=15)	1 (6.7)	4 (26.7)	2 (13.3)	8 (53.3)	
Religion of the respondents					.42
Muslim (n=388)	65 (16.8)	73 (18.8)	76 (19.6)	174 (44.8)	
Non-Muslim (n=12)	0 (0.0)	2 (16.7)	2 (16.7)	8 (66.7)	
Educational status of the respondent					.002
Up to primary (n=76)	15 (19.7)	19 (25.0)	6 (7.9)	36 (47.4)	
Secondary (n=157)	27 (17.2)	34 (21.7)	41 (26.1)	55 (35.0)	
Higher secondary (n=68)	12 (17.6)	12 (17.6)	13 (19.1)	31 (45.6)	
Bachelor’s degree or higher (n=99)	11 (11.1)	10 (10.1)	18 (18.2)	60 (60.6)	
Occupation of the respondent					.24
Housewife (n=347)	60 (17.3)	68 (19.6)	65 (18.7)	154 (44.4)	
Working (n=53)	5 (9.4)	7 (13.2)	13 (24.5)	28 (52.8)	
Family type of the respondent					.98
Nuclear (n=272)	43 (15.8)	51 (18.8)	54 (19.9)	124 (45.6)	
Joint (n=128)	22 (17.2)	24 (18.8)	24 (18.8)	58 (45.3)	
Number of family members					.93
<5 persons (n=193)	30 (15.5)	38 (19.7)	36 (18.7)	89 (46.1)	
≥5 persons (n=207)	35 (16.9)	37 (17.9)	42 (20.3)	93 (44.9)	
Monthly family income (BDT)^b^					.04
≤20,000 (n=143)	30 (21.0)	30 (21.0)	28 (19.6)	55 (38.5)	
20,001‐40,000 (n=157)	22 (14.0)	31 (19.7)	35 (22.3)	69 (43.9)	
≥41,001 (n=100)	13 (13.0)	14 (14.0)	15 (15.0)	58 (58.0)	

a χ2/Fisher exact test significant level.

b A currency exchange rate of 101.85 BDT=US $1 is applicable.

### Variation in Knowledge and Practices of the Respondents

A significant difference in respondents’ knowledge and practices with sociodemographic characteristics was observed (Table 4). The analysis found that the knowledge was comparatively higher among mothers of higher age groups compared to lower age groups (mean knowledge score: 12.13, 95% CI 10.73-13.54 vs 11.23, 95% CI 10.85-11.58; *P*=.01). Similarly, both the knowledge and practice behaviors were significantly higher among mothers with higher education and income than their counterparts. In addition, working mothers and mothers with small families had significantly higher knowledge (Table 4).

**Table 4.** Knowledge and practice variation of mothers according to sociodemographic characteristics.

Characteristics		Knowledge score (range 1-15), mean (95% CI)	*P* value^a^	Practice score (range 1-13), mean (95% CI)	*P* value^a^
Age group (years)			.01		.21
21‐30 (n=209)		11.21 (10.85‐11.58)		6.13 (5.92‐6.35)	
31‐40 (n=176)		11.93 (11.56‐12.29)		6.36 (6.09‐6.62)	
41‐48 (n=15)		12.13 (10.73‐13.54)		6.8 (5.67‐7.93)	
Religion			.22		.19
Muslim (n=388)		11.54 (11.28‐11.80)		6.24 (6.07‐6.41)	
Non-Muslim (n=12)		12.25 (10.53‐13.97)		6.83 (6.08‐7.59)	
Educational status			<.001		<.001
Up to primary (n=76)		9.66 (8.95‐10.37)		6.01 (5.63‐6.40)	
Secondary (n=157)		11.32 (10.97‐11.67)		6.01 (5.75‐6.27)	
Higher secondary (n=68)		12.26 (11.71‐12.82)		6.19 (5.79‐6.59)	
Bachelor’s degree or higher (n=99)		12.93 (12.55‐13.31)		6.88 (6.54‐7.22)	
Occupation			.03		.12
Housewife (n=347)		11.45 (11.17‐11.73)		6.21 (6.02‐6.39)	
Working (n=53)		12.30 (11.72‐12.89)		6.59 (6.19‐6.98)	
Family type			.13		.88
Nuclear (n=272)		11.7 (11.39‐12.00)		6.25 (6.05‐6.45)	
Joint (n=128)		11.28 (10.81‐11.75)		6.28 (5.98‐6.59)	
Number of family members			<.001		.95
<5 persons (n=193)		11.96 (11.6‐12.32)		6.27 (6.03‐6.51)	
≥5 persons (n=207)		11.19 (10.84‐11.55)		6.25 (6.01‐6.48)	
Monthly family income (BDT)^b^			<.001		.002
≤20,000 (n=143)		10.92 (10.48‐11.36)		5.96 (5.68‐6.24)	
20,001‐40,000 (n=157)		11.56 (11.17‐11.95)		6.20 (5.96‐6.45)	
≥40,001 (n=100)		12.49 (12.0‐12.98)		6.77 (6.40‐7.14)	

a Mann-Whitney *U* test and Kruskal-Wallis 1-way ANOVA test.

b A currency exchange rate of 101.85 BDT=US $1 is applicable.

### Association Between Mothers’ Oral Hygiene Knowledge and Practice Levels

**Figure 2.**
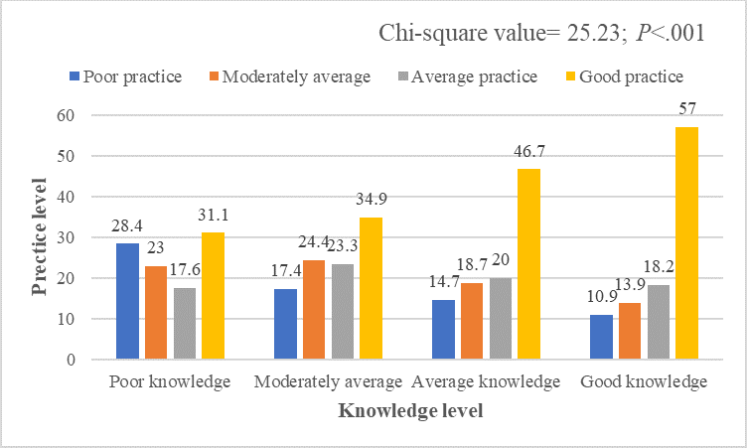
Association between mothers’ knowledge level and practice level.

Figure 2 represents the association between mothers’ oral hygiene knowledge and practice levels. Over 50% of mothers with good knowledge had good practice behaviors regarding their children’s oral hygiene. The Pearson correlation coefficient analysis also found a significant and positive association (*r*=0.301; *P*<.001) between the knowledge and practice scores of the respondents (Multimedia Appendix 6).

## Discussion

### Principal Findings

Oral health is an integral component of overall health, and it is important in our everyday lives. This study intended to evaluate mothers’ knowledge and practices regarding their children’s oral hygiene. An increased knowledge level was observed among older mothers, those with higher education levels, working mothers, and mothers from higher income groups. Similarly, good practices regarding children’s oral hygiene were associated with the mother’s education level and economic status.

### Comparison to Prior Work

To maintain oral health, brushing twice a day is standard [30]. The study found that most mothers know the standard brushing recommendation for their children. Many mothers also agreed that gingival disease was the most common cause of gum bleeding, and brushing and flossing could protect against bleeding gums. The findings align with the existing literature [31]. If one wants to protect themselves against any kind of dental sickness, brushing regularly is required [32]. Over 50% of the mothers in our study agreed with this statement, which is comparable to existing research findings [33]. In this study, less than half of the mothers had good knowledge regarding their children’s oral hygiene, and nearly 1 in every 5 mothers had poor knowledge. The findings suggest that health education programs among mothers regarding their children’s oral hygiene are needed. Various education and awareness programs, including television, social and mass media campaigns, and community-based educational interventions may improve mothers’ knowledge regarding children’s oral hygiene [34-36].

In this study, the mother’s knowledge regarding their children’s oral hygiene was significantly associated with their age, and mothers in the higher age group had comparatively higher knowledge than those in the lower age group. The finding is comparable to many studies that suggest oral health educational programs for younger mothers [34,37,38]. The mother’s educational status and monthly family income were two important predictors for increasing their children’s oral hygiene knowledge and practices. Parents with higher education were more aware of their children’s dental health [39,40]. Our research results align with the existing literature that indicates that mothers who have attained a university degree possess superior knowledge about oral health in comparison to those with a lower level of education [41]. This might be rationalized by the deduction that women with a lower level of education may lack awareness about the consequences of probable risk factors linked to the progression of oral disorders. Consequently, health awareness and promotion play a vital role for mothers who have inadequate educational backgrounds [40,42,43]. Our results align with the existing research, which demonstrates that mothers with extensive knowledge tend to promote good oral health habits in their children [25].

### Strengths and Limitations

This study aimed to identify the variables that impact oral hygiene habits among mothers and evaluate their level of knowledge and compliance with oral hygiene practices. The primary merit of this study is the results. We identified the variables that influence individuals’ understanding and behaviors related to oral hygiene. We experienced a few limitations during this study. First, this was cross-sectional research, which lacks strength in cause-effect analysis. Second, the study was conducted among mothers visiting tertiary-level hospitals in Dhaka. Therefore, there is a chance of nonresponse bias due to convenience sampling.

### Future Directions

Maintaining good oral hygiene is crucial for every child’s overall health; mothers, in particular, play a vital role in this regard. Based on our study findings, the following recommendations may help enhance maternal knowledge and improve children’s oral hygiene practices.

#### Educational Workshops and School-Based Initiatives

Community-based educational programs including workshops and seminars may help educate mothers of different age groups [34,40]. These workshops should focus on the importance of oral hygiene, practical tips for maintaining children’s oral health, and common misconceptions. Monthly informational sessions on oral hygiene practices facilitated by dental health professionals and community health centers could play an important role in improving children’s oral hygiene practices. Various school-based initiatives, like partnering with schools to offer regular seminars and distributing informative materials to parents during parent-teacher meetings that emphasize the critical role of oral hygiene from an early age, could be implemented [37].

#### Incorporate Oral Health Education Into the Curriculum

Integration of basic oral health education into the curriculum of early childhood education programs, ensuring that children learn about oral hygiene from a young age, may help children improve their oral hygiene practices [39,44]. Various programs within schools that encourage parental involvement in learning about and practicing good oral hygiene, and providing resources and support for mothers to reinforce these practices at home may help children improve their oral hygiene practices [44].

#### Media and Technology Use

Launching social media campaigns targeting mothers; using platforms like Facebook, Instagram, and YouTube to disseminate information on children’s oral hygiene; and featuring engaging content such as infographics, videos, and interactive question-and-answer sessions with dental professionals could also be influential initiatives [35].

#### Research and Monitoring

Support should also be provided for ongoing research to monitor the effectiveness of these initiatives and to identify new trends and needs related to children’s oral hygiene [45]. Establishing feedback mechanisms, such as surveys and focus groups, can help gather insights from mothers on the effectiveness of current programs and identify areas for improvement.

### Conclusion

This study revealed that mothers’ knowledge and practices regarding their children’s oral health were insufficient. The mother’s age, education level, family size, and monthly income significantly influenced their knowledge level. Children’s oral hygiene habits were significantly associated with family income and the mother’s educational status. Women aged 41-48 years with a bachelor’s degree or higher, from higher socioeconomic backgrounds, and with school-aged children demonstrated significantly higher levels of knowledge. Mothers with higher socioeconomic status and more education demonstrated a much higher level of dental hygiene practices for their children. The mother’s knowledge regarding their children’s oral hygiene had positive effects and significantly correlated with their children’s oral hygiene practices. The findings of this study emphasize the need for educational and school-based initiatives, accessible dental care services, oral health education in the curriculum, media and technology involvement in oral health educational campaigns, and proper research and monitoring.

## Supplementary material

10.2196/59379 Multimedia Appendix 1 STROBE (Strengthening the Reporting of Observational Studies in Epidemiology) statement: checklist of items that should be included in reports of cross-sectional studies.

10.2196/59379 Multimedia Appendix 2 List of variables used to assess mothers’ knowledge regarding their children’s oral hygiene.

10.2196/59379 Multimedia Appendix 3 List of variables used to assess mothers’ practices regarding their children’s oral hygiene.

10.2196/59379 Multimedia Appendix 4 Mothers’ individual knowledge regarding their children’s oral hygiene.

10.2196/59379 Multimedia Appendix 5 Mothers’ individual practices regarding their children’s oral hygiene.

10.2196/59379 Multimedia Appendix 6 Correlation between knowledge and practice scores.

## Abbreviations

ECC: early childhood caries
NIPSOM: National Institute of Preventive and Social Medicine
STROBE: Strengthening the Reporting of Observational Studies in Epidemiology
