# Supplementary material for: Mothers’ Knowledge of and Practices Toward Oral Hygiene of Children Aged 5-9 Years in Bangladesh: Cross-Sectional Study
Source: JMIRx Med. 2025 Feb 3;6:e59379. doi: 10.2196/59379 (PMC11809941; doi:10.2196/59379)
Supplement: Multimedia Appendix 2 [file xmed-v6-e59379-s002.docx]

Supplementary Table S2. List of variables used to assess mothers’ knowledge regarding their children’s oral hygiene

| ***Knowledge related information (We considered the code ‘1’ for correct answer and ‘0’ for incorrect answer)*** | |
| --- | --- |
| Do you know the importance of brushing teeth? | No=0, Yes=1 |
| What is the recommended frequency of teeth brushing? | No=0, Yes=1 |
| What is the appropriate time of cleaning teeth? | No=0, Yes=1 |
| What is the appropriate duration of cleaning teeth? | No=0, Yes=1 |
| Do you know about different types of toothpaste available for children? | No=0, Yes=1 |
| Do you know the importance of tongue cleaning? | No=0, Yes=1 |
| Do you think gingival disease is the most common cause of gum bleeding? | No=0, Yes=1 |
| Do you think teeth brushing and flossing protect against bleeding gum? | No=0, Yes=1 |
| Do you know about teeth plaque? | No=0, Yes=1 |
| Do you know bacteria can be transmitted from mother to her child if same utensils are used? | No=0, Yes=1 |
| Does sugary diet cause dental caries? | No=0, Yes=1 |
| Does soft drink cause dental caries? | No=0, Yes=1 |
| Does teeth brushing protect against dental caries? | No=0, Yes=1 |
| Does the use of fluoride toothpaste protect against dental caries? | No=0, Yes=1 |
| Is general health affected by dental health? | No=0, Yes=1 |
