# Supplementary material for: Mothers’ Knowledge of and Practices Toward Oral Hygiene of Children Aged 5-9 Years in Bangladesh: Cross-Sectional Study
Source: JMIRx Med. 2025 Feb 3;6:e59379. doi: 10.2196/59379 (PMC11809941; doi:10.2196/59379)
Supplement: Multimedia Appendix 3 [file xmed-v6-e59379-s003.docx]

Supplementary Table S3. List of variables used to assess mothers’ practices regarding their children’s oral hygiene

| ***Mothers' follow-up of practice related information (We considered the code ‘1’ for correct answer and ‘0’ for incorrect answer)*** | |
| --- | --- |
| Does your child brush his/her teeth regularly? | No=0, Yes=1 |
| How many times does your child brush his/her teeth? | Once =0, More than twice=0, Twice =1 |
| How long does your child take to complete one brushing session? | More than 3 mins=0, less than 2 mins=0, 2-3 mins=1 |
| Which aids do your child use for teeth cleaning? | Finger =0, twig=0, Tooth brush=1, Any other=0 |
| Which brushing motion does your child use to brush his/her teeth? | Doesn’t matter=0, Scrubing to and fro=0, Circular=1 |
| When do you change your child’s toothbrush? | No=0, Yes=1 |
| Does your child use toothpaste for cleaning teeth? | No =0, Yes=1 |
| Are your children’s teeth brushed using a toothpaste containing fluoride? | No =0, Yes=1, Don’t know=0 |
| Do you use dental floss for your child? | No=0, Yes=1 |
| Does your child clean tongue? | No=0, Yes=1 |
| Does your child rinse the mouth after eating/drinking? | No =0, Yes=1 |
| At what time do you give the sugary food items to your child? | In between meals=0, Before going to bed=0, Not particular=0, With meals =1 |
| How often do you take your child to the dentist? | No visit=0, Only during problems=0, Every 1 year=0, Every 6 months=1 |
