# Supplementary material for: Mothers’ Knowledge of and Practices Toward Oral Hygiene of Children Aged 5-9 Years in Bangladesh: Cross-Sectional Study
Source: JMIRx Med. 2025 Feb 3;6:e59379. doi: 10.2196/59379 (PMC11809941; doi:10.2196/59379)
Supplement: Multimedia Appendix 4 [file xmed-v6-e59379-s004.docx]

Supplementary Table S4. Individual distribution of mothers’ knowledge regarding their children’s oral hygiene

| **Knowledge related variables** | **Incorrect response**  **f (%)** | **Correct response**  **f (%)** |
| --- | --- | --- |
| Importance of brushing teeth | 19(4.7) | 381(95.3) |
| Recommended frequency of tooth brushing | 71(17.7) | 329(82.3) |
| Appropriate time of cleaning teeth | 77(19.2) | 323(80.8) |
| Appropriate duration of cleaning teeth | 282(70.5) | 118(29.5) |
| Different types of toothpaste available | 76(19.0) | 324(81.0) |
| Importance of tongue cleaning | 35(8.7) | 365(91.3) |
| Gingival disease is the most common cause of gum bleeding | 114(28.5) | 286(71.5) |
| Tooth brushing and flossing protect against bleeding gum | 157(39.2) | 243(60.8) |
| Meaning of teeth plaque | 38(9.5) | 362(90.5) |
| Bacteria transmission from mother to child if same utensils are used | 86(21.5) | 314(78.5) |
| Sugary diet causes dental caries | 13(3.2) | 387(96.8) |
| Soft drink causes dental caries | 105(26.2) | 295(73.8) |
| Tooth brushing protects against dental caries | 20(5.0) | 380(95.0) |
| Fluoride toothpaste protects against caries | 246(61.5) | 154(38.5) |
| Dental health affects general health | 36(9.0) | 364(91.0) |
