# Supplementary material for: Mothers’ Knowledge of and Practices Toward Oral Hygiene of Children Aged 5-9 Years in Bangladesh: Cross-Sectional Study
Source: JMIRx Med. 2025 Feb 3;6:e59379. doi: 10.2196/59379 (PMC11809941; doi:10.2196/59379)
Supplement: Multimedia Appendix 5 [file xmed-v6-e59379-s005.docx]

Supplementary Table S5. Mothers’ individual practices regarding their children’s oral hygiene

| **Practice related variables** | **Incorrect response**  **f (%)** | **Correct response**  **f (%)** |
| --- | --- | --- |
| Regularity of tooth brushing | 19(4.7) | 381(95.3) |
| Frequency of brushing teeth | 223(55.7) | 177(44.3) |
| Duration of brushing teeth | 281(70.2) | 119(29.8) |
| Aids used for teeth cleaning | 4(1.0) | 396(99.0) |
| Brushing method used | 190(47.5) | 210(52.5) |
| Duration of changing tooth brush | 152(38.0) | 248(62.0) |
| Use of toothpaste | 9(2.2) | 391(97.8) |
| Using fluoride toothpaste | 360(90.0) | 40(10.0) |
| Use of dental floss | 389(97.2) | 11(2.8) |
| Do tongue cleaning | 232(58.0) | 168(42.0) |
| Rinse mouth after eating/drinking | 89(22.2) | 311(77.8) |
| Time of giving sugary food items | 350(87.5) | 50(12.5) |
| Taking to dentist | 399(99.7) | 1(0.3) |
