# Supplementary material for: Mothers’ Knowledge of and Practices Toward Oral Hygiene of Children Aged 5-9 Years in Bangladesh: Cross-Sectional Study
Source: JMIRx Med. 2025 Feb 3;6:e59379. doi: 10.2196/59379 (PMC11809941; doi:10.2196/59379)
Supplement: Multimedia Appendix 6 [file xmed-v6-e59379-s006.docx]

Supplementary result S6: Correlation between knowledge and practice score

|  |  | Knowledge score | Practice score |
| --- | --- | --- | --- |
| Knowledge score | Pearson Correlation | 1 | .296^**^ |
|  | P value |  | 0.0001 |
| Practice score | Pearson Correlation | .296^**^ | 1 |
|  | P value | 0.0001 |  |
| **. Correlation is significant at the p<0.01 level (2-tailed). | | | |
